# Spatial Segregation between Invasive and Native Commensal Rodents in an Urban Environment: A Case Study in Niamey, Niger

**DOI:** 10.1371/journal.pone.0110666

**Published:** 2014-11-07

**Authors:** Madougou Garba, Ambroise Dalecky, Ibrahima Kadaoure, Mamadou Kane, Karmadine Hima, Sophie Veran, Sama Gagare, Philippe Gauthier, Caroline Tatard, Jean-Pierre Rossi, Gauthier Dobigny

**Affiliations:** 1 Centre Régional Agrhymet, Département Formation Recherche, Niamey, Niger; 2 Université Abdou Moumouni, Faculté des Sciences, Niamey, Niger; 3 Direction Générale de la Protection des Végétaux, Ministère de l′Agriculture, Niamey, Niger; 4 IRD, Aix Marseille Université, LPED (UMR IRD-AMU), Marseille, France; 5 IRD, CBGP (UMR IRD-INRA-Cirad-SupAgro Montpellier), Campus International de Baillarguet, Montferrier-sur-Lez, France; 6 Centre Régional Agrhymet, USAid/Fews-Net, Niamey, Niger; 7 IRD, CBGP, Campus ISRA-IRD de Dakar-Bel-Air, Dakar, Senegal; 8 INRA, CBGP, Campus International de Baillarguet, Montferrier-sur-Lez, France; Linneaus University, Sweden

## Abstract

Invasive rodents have been responsible for the diffusion worldwide of many zoonotic agents, thus representing major threats for public health. Cities are important hubs for people and goods exchange and are thus expected to play a pivotal role in invasive commensal rodent dissemination. Yet, data about urban rodents' ecology, especially invasive vs. native species interactions, are dramatically scarce. Here, we provide results of an extensive survey of urban rodents conducted in Niamey, Niger, depicting the early stages of rodent bioinvasions within a city. We explore the species-specific spatial distributions throughout the city using contrasted approaches, namely field sampling, co-occurrence analysis, occupancy modelling and indicator geostatistics. We show that (i) two species (i.e. rural-like vs. truly commensal) assemblages can be identified, and that (ii) within commensal rodents, invasive (*Rattus rattus* and *Mus musculus*) and native (*Mastomys natalensis*) species are spatially segregated. Moreover, several pieces of arguments tend to suggest that these exclusive distributions reflect an ongoing native-to-invasive species turn over. The underlying processes as well as the possible consequences for humans are discussed.

## Introduction

Since the last decades, urbanization has been dramatically increasing all over the World: while 29% of humanity lived in cities in 1950, ∼53% is currently urban, and 67% will be urban by 2050 [1]. Sub-Saharan Africa is currently the most rapidly urbanizing continent, with more than 80 urban centers predicted to reach one million inhabitants by 2025 [1]. Niamey, main town of Niger and focus of the present study, is no exception: for the period 2005–2010, its agglomeration has ranked 23 in the World and 5 in Africa for the average annual rate of rural-to-urban change (5.99%), and it is expected to rank 12 and 4, respectively, for the period 2010–2015 [1]. Of course, this is accompanied by an explosive demographic growth, with ∼34,000 inhabitants in 1960 up to 650,000 and >1,200,000 in 2000 and 2010, respectively [2].

Urbanization represents an extreme situation along the gradient of human-mediated modification of the environment, usually accompanied by drastic changes in abiotic (e.g., soil substrate, hydrographic networks, atmosphere composition, etc) and biotic (e.g., species diversity, abundance and distribution) factors [3]. Indeed, it induces dramatic changes in both landscape and human activities that, in turn, deeply impact the composition and structure of biodiversity within cities. In particular, human infrastructures coupled with permanent supply of a wide range of resources provide a compelling advantage to opportunistic and highly competitive species that display important adaptation abilities [4], [5]. Invasive species are expected to show such characteristics. Moreover, cities constitute major crossroads for people and goods exchange, thus increasing the chance of the involuntary and repeated introductions of alien organisms and their subsequent spread out. Altogether, this makes urban areas particularly prone to bioinvasions.

Commensal rodents such as rats (*Rattus norvegicus*, *R. rattus*, *R. tanezumi*, *R. exulans*) and mice (*Mus musculus*) constitute major invaders, with the house mouse *M. musculus* and the black rat *R. rattus* being listed among the 100 “worst invasive alien species in the World” [6]. These species have settled on all continents [7] following human migrations and trade (e.g., [8]–[13]). They may live in close proximity to human beings and they have become the only remaining commensal rodent species in many cities throughout the World. Beyond the threat for local biodiversity, this may have major consequences for public health [14] since rats and mice are involved in the maintaining and circulation of a wide range of human pathogens (review in [15]). Consequently, introduction of new rodent reservoirs as well as potentially new rodent-borne pathogens may deeply impact host/parasites communities and interactions, hence potentially favouring disease (re)emergence, especially in cities where human/rodent interactions are expected to be favoured [14].

Yet, our knowledge about urban rodent ecology is rather low in regards to the societal stake. Some authors have documented the rodent species assemblages (both native and invasive) and their distribution and/or preferred habitat within urban and peri-urban habitats (e.g., [5], [16]–[20]). Others have focused on the role of invasive species in the epidemiology of pathogens within a given town (e.g., [21]–[22]). Finally, studies have been conducted for a better understanding of invasive rodents' urban ecology (e.g., black and Norway rats: reviewed in [23]), with some very rare instances where the interactions with native species is taken into account (e.g., [24]). As far as we know, no study has ever been performed to specifically investigate the respective distribution of invasive *vs*. native rodent species within the urban environment, especially in the context of an ongoing invasion. Here we present a case-study in the young city of Niamey (it was created *ex nihilo* by the French colonizers at the very end of the 19^th^ century; [25]–[26]) which constitutes an invasion front for the black rat *R. rattus* and the house mouse *Mus domesticus* [27]. We describe the urban rodent community on the basis of extensive field trapping campaigns, and then further compare the species-specific distributions of commensal taxa (with special emphasis on native vs. invasive respective spatial ranges) using independent approaches (co-occurrence analysis, occupancy modelling and indicator geostatistics). Finally, we discuss the underlying processes and the potential implications in terms of rodent control and public health.

## Materials and Methods

### Trapping

**Figure 1:**
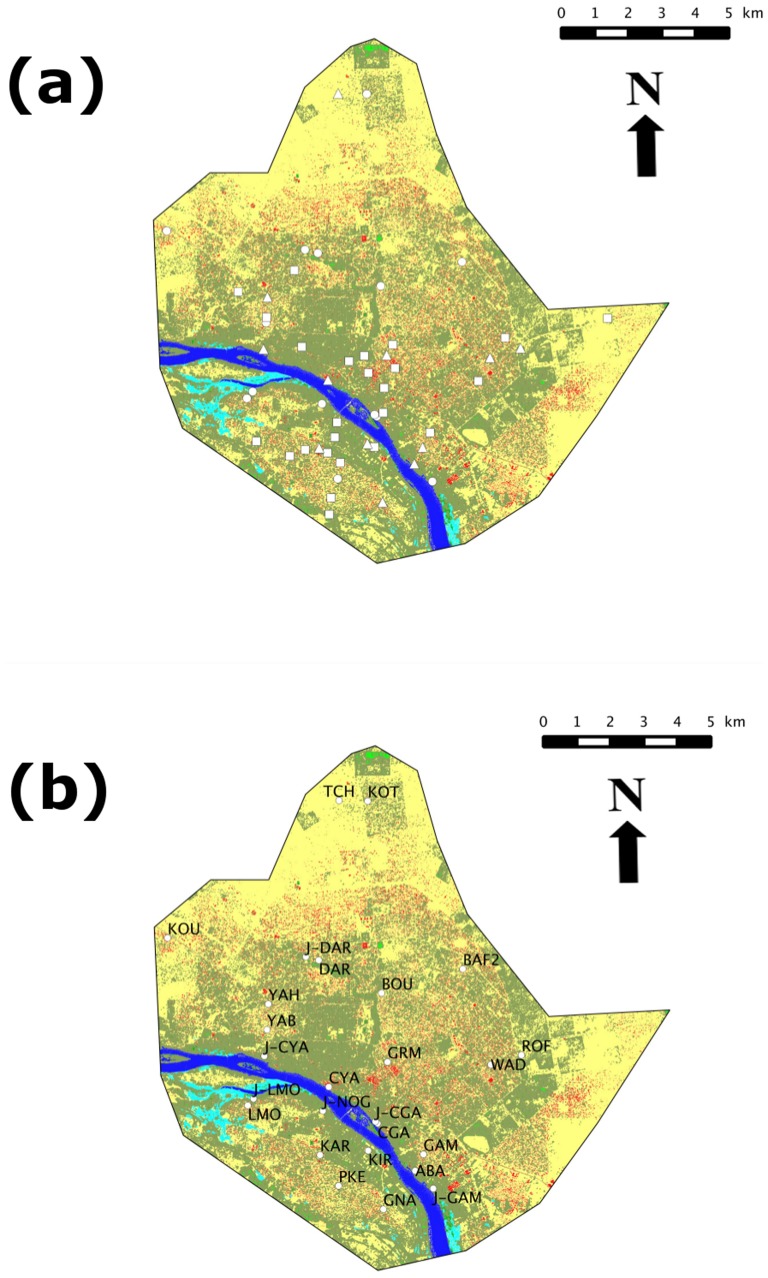
All trapping investigated localities within the city of Niamey (a): circles, squares and triangles indicate sites investigated through SP, OP and SP+OP protocols, respectively. Mapping of SP localities only, with their acronyms referring to Table 1 (b). On both maps, colors of the background correspond to GIS-based categories of landscape elements: yellow for non-covered soil; red for buildings; dark and light green for trees and diverse vegetation, respectively; dark and light blue for Niger River and other water surfaces, respectively.

In the present paper, we design as “localities” the areas of the city that correspond to districts of Niamey (e.g., Lamordé, Koubia, etc; see Figure 1b and Table 1). “Sites” refer to more precisely localized places within localities where trapping was performed (e.g., households, gardens, shops, stores, industrial complexes, etc). Field work was conducted between October 2009 and February 2011. Taking into account the important trapping effort that was required to sample the 215 sites investigated within Niamey (see below), it was not realistic to consider studying diachronic processes. As a consequence, we made the *ad hoc* hypothesis that our 18 months-long survey provides a temporal ‘snap-shot’ of the rodent distributions in the city. Two different and complementary sampling protocols were conducted in parallel. The first one, hereafter referred as to the standardized protocol (SP), consisted in trapping sessions using comparable procedures set up in different localities. The second one, referred as to “opportunistic protocol” (OP), corresponded to punctual trapping sessions that were organized either to complement our SP-based sampling, to obtain a geographically more diverse sampling, and/or to address people complaints about pest rodents in their gardens or houses. Figure 1a shows the spatial distribution of the sampling effort using SP, OP or both.

**Table 1:** Trapping efforts and results, environment type, number of sites and GPS coordinates in all localities investigated using the standardized (SP) and opportunistic (OP) protocols.

Locality	Environment	Protocol	SP		OP	GPS coordinates		Rodent species captured						
	type		Number sites	Trap nights	Trap nights	Lat (N)	Long (E)	Mna	Rra	Mmu	Ani	Cga	Tgr	Nha
ABA	ind	SP+OP	1	478	412	13.4895	2.12275	0	77	0	0	0	0	0
BAF2	hab	SP	10	370	-	13.54401	2.1357	33	0	0	0	0	0	0
BAN	hab	OP	-	-	n/a	13.52161	2.1167	0	1	0	0	0	0	0
BOU	hab	SP	8	449	-	13.53742	2.11331	47	0	0	0	0	0	0
CGA	hab	SP	9	326	-	13.50222	2.11235	1	19	0	0	0	0	0
COA	pb	OP	-	-	8	13.53571	2.07399	2	0	0	0	0	0	0
CRA-1	fal	OP	-	-	n/a	13.49235	2.09877	0	0	0	0	3	0	0
CRA-2	fal	OP	-	-	>262	13.49655	2.10079	0	0	0	0	5	2	1
CRA-3	fal	OP	-	-	n/a	13.5006	2.10141	0	0	0	4	0	0	0
CYA	hab	OP+SP	14	500	>8	13.51204	2.09884	65	4	0	0	0	0	0
DAR	hab	SP	8	531	-	13.54624	2.09594	40	0	0	0	0	0	0
GAM	hab	OP+SP	13	452	n/a	13.49392	2.12501	24	0	0	0	0	0	0
GAM-1	hab	OP	-	-	2	13.49792	2.12705	2	0	0	0	0	0	0
GAW	hab	OP	-	-	36	13.4897	2.10232	6	0	0	0	0	0	0
GNA	hab	OP+SP	11	400	n/a	13.47908	2.11402	29	0	0	0	0	0	0
GOU	hab	OP	-	-	56	13.51856	2.10883	0	1	8	0	0	0	0
GRM	hab	OP+SP	7	305	n/a	13.51882	2.115	0	7	63	0	0	0	0
GRM-M	mkt	OP	-	-	80	13.51527	2.11732	0	4	0	0	0	0	0
HPO	pb	OP	-	-	80	13.50992	2.11438	0	4	1	0	0	0	0
J-CGA	gar	SP	1+1 build.	208	-	13.50271	2.11174	0	0	0	0	0	0	0
J-CYA	gar	OP+SP	3+3 build.	661	>510	13.52029	2.08104	0	19	0	3	0	0	0
J-DAR	gar	SP	4+1 build.	320	-	13.54714	2.09238	0	0	0	5	0	0	0
J-GAM	gar	SP	2+2 build.	480	-	13.48473	2.12775	1	0	0	13	0	0	0
J-KIR1	gar	OP	-	-	n/a	13.49397	2.1117	8	0	0	4	0	0	0
J-KIR2	gar	OP	-	-	n/a	13.47573	2.09936	0	0	0	0	2	0	0
J-LMO	gar	SP	4+4 build.	320	-	13.5088	2.0781	0	0	0	19	2	0	0
J-NOG	gar	SP	3	212	-	13.50558	2.09723	0	0	0	22	0	0	0
KAR	hab	OP+SP	18	707	>583	13.49366	2.0965	76	0	0	0	0	0	0
KAR-1	hab	OP	-	-	n/a	13.49143	2.08843	12	0	0	0	0	0	0
KAR-2	hab	OP	-	-	70	13.49316	2.09262	12	0	0	0	0	0	0
KIR	ind	OP+SP	1	381	705	13.49489	2.10978	0	24	0	0	0	0	0
KIR-1	hab	OP	-	-	20	13.48022	2.09984	5	0	0	0	0	0	0
KOT	hab	SP	7	266	-	13.58922	2.10928	10	0	0	0	0	0	0
KOU	hab	SP	12	378	-	13.55207	2.05424	26	0	0	0	0	0	0
KOU-1	hab	OP	-	-	22	13.56106	2.04155	3	0	0	0	0	0	0
LMO	hab	SP	7	418	-	13.50696	2.07653	36	0	0	0	0	0	0
NPO	pb	OP	-	-	25	13.51709	2.10466	0	2	0	0	0	0	0
PEM	mkt	OP	-	-	374	13.51396	2.10997	24	13	0	0	0	0	0
PGP	pb	OP	-	-	8	13.52093	2.09161	0	1	0	0	0	0	0
PKE	hab	SP	9	448	-	13.48536	2.10164	40	0	0	0	0	0	0
REC	hab	OP	-	-	n/a	13.54157	2.0895	2	0	0	0	0	0	0
RFN	hab	OP	-	-	34	13.52893	2.17587	5	0	0	0	0	0	0
ROF	hab	OP+SP	4	370	24	13.52081	2.15193	23	0	0	0	0	0	0
ROF-1	hab	OP	-	-	n/a	13.52358	2.14766	1	0	0	0	0	0	0
RTO	ind	OP	-	-	270	13.49539	2.07916	15	0	0	0	0	0	0
TCH	hab	OP+SP	5	228	50	13.58936	2.10137	22	0	0	0	0	0	0
TER	hab	OP	-	-	n/a	13.50323	2.11413	0	1	0	0	0	0	0
WAD	hab	OP+SP	7	497	>48	13.5182	2.14351	13	0	0	0	0	0	0
WAD-1	coa	OP	-	-	>48	13.51186	2.14032	0	1	0	0	0	0	0
YAB	hab	SP	12	449	-	13.5274	2.08175	27	0	0	0	0	0	0
YAB-1	hab	OP	-	-	10	13.52891	2.08186	10	0	0	0	0	0	0
YAH	hab	OP+SP	9	484	120	13.53435	2.08208	28	0	0	0	0	0	0

“Ind”, “hab”, “gar”, “coa” and “mkt” stand for industrial site, habitations, gardens, coach station and market, respectively. “Mna”, “Rra”, “Mmu”, “Ani”, “Cga”, “Tgr” and “Nha” correspond to *Mastomys natalensis*, *Rattus rattus*, *Mus musculus*, *Arvicanthis niloticus*, *Cricetomys gambianus*, *Taterillus gracilis* and *Nannomys hausa*, respectively.

Both Sherman and locally made wire-mesh traps were used and baited using a mixture of nut butter and ‘soumbala’ (local spice made from the néré tree, *Parkia biglobosa*). All sites were geolocalized using a Garmin 12XL GPS. A satellite image of Niamey was obtained as a part of a Spot Image (scene reference number 506 132 308 121 010 151 32 T, CNES 2008 ©) and was used as a background for our figures.

Details about localities as well as respective trapping efforts and results are provided in Table 1.

The whole trapping campaign was validated by national and local authorities (scientific partnership agreement number 301027/00 between IRD and the Republic of Niger). At the French level, all sampling procedures were conducted by biologists from the CBGP holding certificates to carry out experiments on live animals (agreements number C34-106 and C34-169-1, valid until 16^th^ December 2016 and 25^th^ July 2017, respectively). None of the rodent species investigated in the present study has protected status (see UICN and CITES lists). All animals were treated in a humane manner in accordance with guidelines of the American Society of Mammalogists. All rodents were euthanized through cervical dislocation. Permit to enter and work within private properties were systematically obtained through oral but explicit agreement from adequate institutional (research agreement quoted above; mayor) and traditional authorities (both neighbourhood and family chiefs).

#### Standardized protocol (SP)

In total, 26 localities (Table 1 and Figure 1) were investigated within Niamey using the SP procedure: 18 habitations districts (BAF2, BOU, DAR, CGA, CYA, GAM, GNA, GRM, KAR, KOT, KOU, LMO, PKE, ROF, TCH, WAD, YAB and YAH), 7 intra-city cultivated gardens (J-CGA, J-CYA, J-DAR, J-GAM, J-LMO and J-NOG) and 2 industrial zones (ABA and KIR). Between 4–18 sites were sampled in each locality, thus reaching a total of 189 SP sites for the whole town (Table 1), 170 of them corresponding to habitations or familial ventures (e.g., stores, sewing of repair shops, etc).

In gardens, only wire-mesh traps were used along lines with a 5–10 meters inter-trap space. In the core city, a maximum of compartments (rooms within buildings, yards, external store rooms, etc) was investigated per site, with both Sherman and wire-mesh traps being used conjointly in order to limit potential bias due to the type of traps.

All sites were investigated during four consecutive nights, with each trap having captured being replaced on the next day. Each trap was individually identified, thus allowing us to precisely monitor which traps was set up, when and where, hence which individual rodents was trapped, when and where. In only three localities (GNA, KAR and PKE) did we conduct the SP survey during two periods. A total of 10,638 night-traps were performed in the framework of the SP, with 7,578, 2,201 and 859 of them concerning habitations districts, gardens and industrial spots, respectively (Table 1).

#### Opportunistic protocol (OP)

Additional trapping sessions were organized in various places of town independently of the SP. They were motivated by assistance to people complaining about rodents, by opportunities to complement samples previously obtained in some SP-investigated localities, or to target poorly surveyed areas of Niamey. This opportunistic protocol (OP) did not follow any formal (i.e., repeated) experimental design, and trapping could last from one single to several consecutive nights. Nevertheless, traps were checked for captures every day and potentially replaced for the subsequent night(s).

Although it was impossible to accurately assess the trapping effort in some OP instances (closed houses, absence of people, etc), a minimum of 3,865 night-traps were performed. In total, 38 localities were explored through the OP (Table 1 and Figure 1a), with 12 of those having already been surveyed using the SP (garden: J-CYA; industrial spots: ABA and KIR; habitation areas: CYA, GAM, GNA, GRM, KAR, ROF, TCH, WAD and YAH). The 26 OP-specific sampled localities correspond to 5 inner-city gardens or fallow lands (CRA-1, CRA-2, CRA-3, J-KIR1 and J-KIR2), 2 markets (PEM and GRM-M), 3 public buildings (HPO, NPO and PGP), 1 industrial store room (RTO), 1 coach station (WAD-1) and 14 additional habitation zones (BAN, COA, GAM-1, GAW, GOU, KAR-1, KAR-2, KIR-1, KOU-1, REC, RFN, ROF-1, TER, WAD and YAB-1).

### Species-specific identifications of rodents

West African rodent genera are usually complex of sibling species (reviewed in [28]). Therefore, a special attention was paid to unambiguous diagnosis of rodents known to belong to taxonomically problematic taxa. To do so, we relied on different approaches depending on the genus considered. Following a recent molecular study [29] showing that giant rats from the Sahel (including specimens from Niamey) all belong to the same species, *Cricetomys* individuals were identified at the species-level on morphological criteria, only. Individuals from the genus *Mus* (N = 6), *Arvicanthis* (N = 4) and *Taterillus* (N = 2) were karyotyped for unambiguous species-specific diagnosis. The only *Mus* subgenus *Nannomys* specimen was identified through complete cytochrome b sequencing following [30]. Species from the genus *Mastomys* are almost impossible to distinguish from each other solely on morphology. Yet, four species are known from West Africa [28], three of which have already been found in Niger [31]–[32]. In the same manner, *R. rattus* and *R. tanezumi* are often difficult to discriminate phenotypically. Although only *R. rattus* is currently known from Western Africa [28], *R. tanezumi* has recently been reported in Southern Africa [12]. Given these difficulties, a special effort was made for the precise taxonomic identification of *Mastomys* and *Rattus* individuals. To do so, 355 *Mastomys* specimens (originating from all SP localities and 118/119 SP sites, as well as all but one OP localities where *Mastomys* were captured; see [27]) were studied using a species-specific RFLP-based test [33]. In addition, 30 specimens were karyotyped (see [32]) and 607 individuals were further genotyped for population genetics purposes using 16 specific microsatellite loci [34]–[35]. In a similar way, all rats were investigated using a panel of 17 microsatellite loci [36]–[37].

### Co-occurrence analyses

In order to investigate whether pairwise species were aggregated, segregated or randomly associated, we compared observed and expected patterns under the null hypothesis of random assembly [38]–[39] within the core-city (i.e., gardens excluded). Input data was a detection/non-detection matrix with species in rows and trapping sites in columns, taking either the two industrial-like ones (i.e., ABA and KIR) into account (N = 172) or not (N = 170). To do so, we relied on the standardized C-score (SCS; [40]) as a quantitative index of co-occurrence, with significant negative and positive SCS indicating aggregation and segregation, respectively. We compared each single SCS of the observed data matrix to values from 30,000 randomly constructed matrices. We used a null model where (i) species occur at the same frequency as in the original dataset (i.e., fixed row total constraint) and (ii) the probability of a species occurring in a given trapping site is directly proportional to the associated sampling effort (i.e., column total constraints with the number of night-traps as site weights). Each trapping site was weighted by its corresponding number of night-traps in order to take sampling effort into account (column total constraint with the number of night-traps as site weights). Separate analyses for each pair of commensal rodent species (namely, *R. rattus*-*M. natalensis*, *R. rattus*-*M. musculus* and *M. musculus*-*M. natalensis*) and randomization tests were conducted with EcoSim v.7 [39].

### Occupancy modelling

In most sampling protocols, detection of an individual is indicative of “presence”, but non detection of the species is not equivalent to absence. As a consequence, estimates of the proportion of sites occupied could be negatively biased to some unknown degree because species can go undetected while present [41], inducing wrong inferences on co-occurrence. To account for the probabilities of not capturing a species although the species is present, we analysed the data of capture following a framework of occupancy [41]. In such framework, *ψ_i_* defines the probability that a species *i* is present at a site and *p_i_* the probability that this species *i* is captured (thus detected) at that same site given presence. *K* night-traps in this site result in a history of capture, that is a series of 1 and 0 coded when a given species is captured or not. For example, assume a site with 2 night-traps; during the first night the species is captured, the second night the trap remains empty. Such site will have a history of capture *h* =  {1,0}. Because the species has been captured the first night, we know it is present in this site but has not been captured during the second night trap. Hence, the probability of the history *h*, Pr(*h*), is simply Pr(*h*)  = ψ_i_*p_i_*(1−*p_i_*). Let's assume another site with a history *h* =  {0,0}. At this site, we do not know if the species is present but not captured, or truly absent. Such an history corresponds to a probability Pr(*h*) = ψ_i_*(1−*p_i_*)*(1−*p_i_*)+(1−*ψ_i_*). All sites can have their history of capture modelled as function of parameters of capture *p_i_* and presence *ψ_i_* resulting in the likelihood of the data observed. *p_i_* and *ψ_i_* can then be estimated by Maximum Likelihood.

We conducted separated occupancy analyses for the one native (*M. natalensis*) and the two invasive (*R. rattus* and *M. musculus*) commensal species within the core city. To do so, we scored the presence/absence of capture for each night-trap in 166 of the 172 SP sites (six sites were not usable due to a misleading monitoring of traps). We then estimated a probability of capturing a given species in a trap, accounting for the impact of the type of traps used (Sherman or locally made wire-mesh traps) into account. Based on these different estimates of probability of capture, we then estimated the conditional probability of presence of a species, that is, the probability of presence conditional on the capture history of a trapping site Pr(*ψ_i_ |h*). If the species has been captured in the site, Pr(*ψ_i_ |h*) = 1; we know the species is present in this site. If the species has not been captured in this particular site, then its probability of presence Pr(*ψ_i_ |h*) can be calculated using Bayes conditional probability [42]: Pr(*ψ_i_ |h*) = Pr(*h|ψ_i_*)*ψ_i_/Pr(*h)*.

Analyses were performed using the software PRESENCE (http://www.mbr-pwrc.usgs.gov/software/presence.html) for model selection following the Akaike criterion as well as for estimating the different parameters of the models in Maximum-Likelihood.

### Indicator geostatistics

The spatial distribution of the two most widely distributed rodent species (*M. natalensis* and *R. rattus*) was studied using geostatistics, a branch of spatial statistics [43]. The other species were too rare to allow a proper geostatistical analysis. The approach developed here is thus spatially explicit since it specifically accounts for the spatial position of each sample.

Let z(u_α_), with α = 1, 2, n, be a set of n values of rodent density measured in the city of Niamey where u_α_ is the vector of spatial coordinates of the 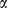 ^th^ observation. In geostatistics, spatial patterns are generally described in terms of dissimilarity between sample location as a function of the distance separating these samples. The average dissimilarity between samples separated by a vector h is quantified by means of the experimental semivariogram or variogram for short 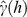, which is computed as half the average squared difference between the datum associated to every data pairs: 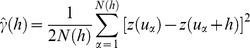



where N(*h*) is the number of data pairs for a given separating distance *h*, z(u_α_) and z(u_α_+h) the observed values at all sampling points separated by a vector *h*.

The shape of the variogram provides important information about the scale and intensity of the spatial structures at hands. Flat variograms indicate a lack of spatial structure, that is, a random spatial distribution. On the contrary, patchy distributions generally lead to variograms exhibiting an initial increase of 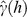 with h until it levels off to a plateau [43].

Geostatistics were originally developed in the field of geology [44], progressively percolated in ecology [45] and are now well documented in various fields of environmental research [43], [46]–[49].

The rodent density strongly fluctuates even at the scale of neighbouring sites and the corresponding variability may be difficult to interpret. It might also lead to highly skewed frequency distributions with possible side effects upon our perception of spatial structures. Since we are here interested in the spatial distribution of and co-occurrence between rodent species, we recoded the species abundance into detection/non-detection data. This corresponds to creating an indicator variable i(u_α_; z_k_) for the threshold z_k_ = 0 defined as: 
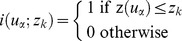



The indicator variogram is then computed by replacing the original density values by the indicator data [50]. It measures the transition frequency between detection and non-detection of the species *s* as a function of h. The greater the semi-variance 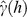, the less connected in space are the detection or non-detection values.

In the same way as for variograms, indicator variograms can be modelled using some authorized functions (e.g., [43]–[44]) and the resulting model used for interpolation purposes by kriging. In this study, we used the ordinary indicator kriging in order to interpolate the probability that the density of species *s* does not exceed the threshold z_k_ across the survey area. This probability is estimated as a linear combination of neighbouring indicator data [43], [51]. We took advantage of this approach to (i) assess spatial structures of rodent occurrences by means of indicator variogram analysis and (ii) map the probability of detection of each species by means of simple ordinary indicator kriging.

Cross-variograms measure the joint variability of two variables, here species z and y. 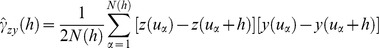



When z and y are indicator-transformed variables, the indicator cross-variogram quantifies how often values separated by a vector h are on opposite sides of the threshold value. The greater the cross semi-variance, the less connected in space are the detection or non-detection values [43]. We used the cross-variogram as a tool to explore the spatial covariation between indicator transformed rodent abundances.

## Results

### Rodent diversity and distribution within Niamey

**Figure 2:**
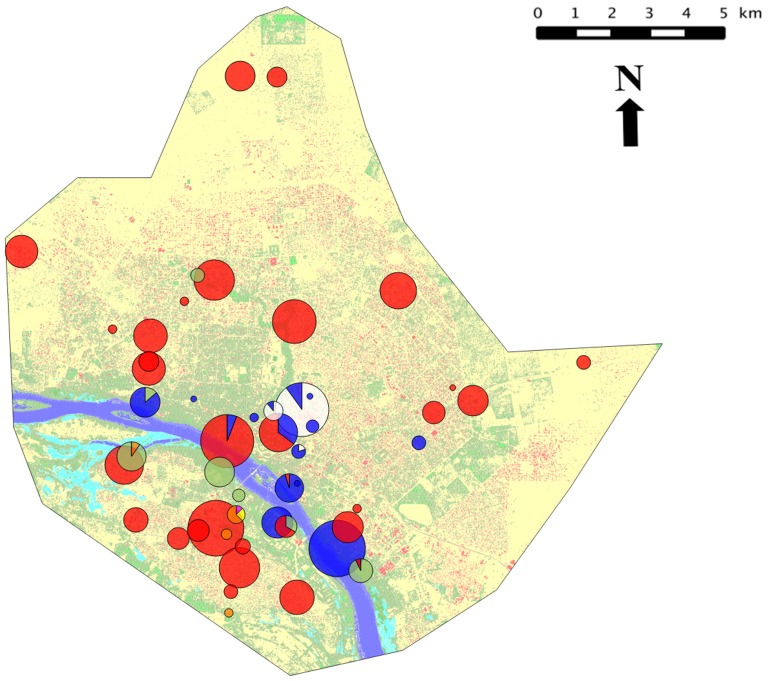
Spatial distribution of each of the seven species captured in Niamey. Each circle corresponds to one OP and/or SP trapping site (see Table 1 and Figure 1). The circle size is proportional to the total number of rodent captures. Each color stands for a given species: red for *Mastomys natalensis*, white for *Mus musculus*, blue for *Rattus rattus*, green for *Arvicanthis niloticus*, orange for *Cricetomys gambianus*, yellow for *Taterillus gracilis* and rose for *Nannomys hausa*.

Rodents were trapped in all but one (J-GAM) of the 52 sampled localities, reaching a total of 987 individuals that belong to seven unambiguously identified species (Figure 2 and Table 1). Among the latter, five were native, i.e., *Cricetomys gambianus* (N = 12), *Arvicanthis niloticus* (N = 70), *Taterillus gracilis* (N = 2), *Nannomys haussa* (N = 1) and *M. natalensis* (N = 648), while two were invasive: *M. musculus* (N = 72) and *R. rattus* (N = 182). An eighth species, namely the African ground squirrel *Xerus erythropus*, was observed in a few instances but never caught due to inappropriateness of the traps used. In addition, 69 shrews were caught in 33 SP sites from 13 SP localities (see [27]): the sequencing of the cytb, CO1 and 16S mitochondrial genes from seven individuals revealed the existence of one species, *Crocidura olivieri* (F. Jacquet et al., pers. comm.).

All *C. gambianus*, *T. gracilis* and *A. niloticus* were found exclusively in gardens, fallow lands and rice fields, the only exception being one *A. niloticus*caught in a store room which, however, lied within a garden. *M. natalensis* were all found associated with typical human infrastructures (houses, traditional shops and/or ventures, store rooms, markets). In no instance could we find this species elsewhere than in such habitats: even those rare individuals found in gardens (N = 9; Table 1) were in fact trapped inside farmers' houses. Interestingly, this strictly commensal species was caught in all habitation localities except one, namely GRM where only *M. musculus* and a few *R. rattus* were captured (Table 1). The latter district is a central and rather poor one that borders one of the main markets of the city. Inside the market itself (GRM-M), only black rats could be trapped. A few other mice were found in two other surrounding localities (Table 1) either in houses (GOU) or in a public building (HPO). On the contrary, *R. rattus* was also present in several other areas of limited extent (Figure 2 and Table 1), essentially in commercial-like and industrial-like zones: two industrial complexes (ABA: slaughter house, and KIR: husking rice industry; N = 101), one additional market (PEM; N = 13), three public buildings (HPO, NPO and PGP; N = 5) and one coach station (WAD-1; N = 5). Only in some rare instances could we find black rats in habitations (BAN, TER, GOU, CGA and CYA; N = 26, 22 of which come from the same two houses in CGA and CYA).

In summary, at least two species (*A. niloticus* and *C. gambianus*) are tightly associated with intra-city but rural-like environments (Figure 2 and Table 1). Although they may belong to the latter assemblage, data about *M. N. hausa*, *T. gracilis* and *X. erythropus* are too scarce to draw any robust picture of their precise distribution (Table 1). In the core city, one native (*M. natalensis*) and two invasive (*R. rattus* and *M. musculus*) species were found and they should be considered as the only really urban and commensal ones in Niamey (Figure 2 and Table 1). Among them, *M. natalensis* is largely dominant, except in three localities (CGA, CYA and PEM) and three trapping sites (two habitations and one market, representing 1.5% of all trapping sites) where it co-exists with black rats (Figure 2 and Table 1). Rats and mice were found together in two trapping sites (HPO and GOU) while only rats were found in several other localities, especially in the industrial ones (ABA and KIR) where they are most probably the only present species. *M. musculus* seems restricted to the central area of the town, and was clearly preponderant in one habitation locality (GRM) although they were caught with rats within two sites. Finally, in no instance could we find mice and *M. natalensis* in the same sites or even localities (Figure 2 and Table 1).

### Co-occurrence analyses and occupancy modeling

These strongly exclusive distribution patterns were also retrieved through the co-occurrence analyses where both *Mastomys* and *Rattus* (all SP trapping sites: SCS  = 5.29, p<0.00001 based on 30,000 iterations; without ABA and KIR: SCS  = 3.86, p = 0.0032), and *Mastomys* and *Mus* (all SP trapping sites: SCS  = 6.38, p<0.00001; without ABA and KIR: SCS  = 6.3, p<0.00001) were found to segregate highly significantly. On the contrary, *Rattus* and *Mus* were found either randomly associated (all SP trapping sites: SCS  = −1.98, p = 0.099) or slightly aggregated (without ABA and KIR: SCS  = −2.89, p = 0.033).

The probability of capture as calculated through occupancy modelling varied strongly among species and between types of traps (see Table S2). Indeed, *M. musculus* had a probability to be captured in a Sherman trap of 0.297 (standard error, SE±0.038) but the species was never captured in a locally made wire-mesh trap. *M. natalensis* had a probability of capture of 0.139 (SE±0.007) in a Sherman, and this value was almost null with a locally made wire-mesh traps (0.016 SE±0.002). *R. rattus* had a low probability of capture for both Sherman and locally made traps (0.040 SE±0.008 and 0.079 SE±0.011, respectively). Despite an overall low probability of capture, the large number of traps per site as well as the concomitant use of both types of traps strongly reduced the likelihood of a species being present but not captured.

The probabilities of detection of the native *M. natalensis*, the invasive *M. musculus* and/or *R. rattus* for each of the 166 SP trapping sites that were investigated through occupancy modelling are provided in Table S1. In essence, the occupancy probabilities (i.e., taking into account the probability of ‘not capturing a species even when present’) showed the same overall pattern of segregated species-specific distributions. Indeed, in 114 SP sites where the probability of *M. natalensis* detection was 100%, the probability of invasive rodents (i.e., *R. rattus* and/or *M. musculus*) never exceeded 3.7%. Conversely, in 5 sites where invasive rodents showed 100% of detection probability, the native species had less than 3.5% of chances to be identified. In 38 sites, *M. natalensis* was detected with a probability of less than 100% but still ≥10% (among which 34 exceeded 0.25%) while invasive rodents had less than 3.2% to be scored. In 7 sites, both native and invasive species displayed very low probabilities of capture (<6.4% and <0.3%, respectively). Finally, in 7 sites, we found maximum detection probabilities of mice and/or rats together with reasonably (18.5%–52.2%; N = 6 sites) to very (100%; N = 1) high *M. natalensis'* ones. These last seven sites correspond to (i) sites where both invasive and native rodents were indeed trapped (see C-CGA-4 in Table S1), (ii) sites where only invasive rodents were captured but which are located in localities where native rodents were also caught (C-CGA-2 and C-CYA-10) or (iii) sites where only mice and rats were observed (C-GRM-2, 3, 4 and 6).

### Indicator geostatistics

**Figure 3:**
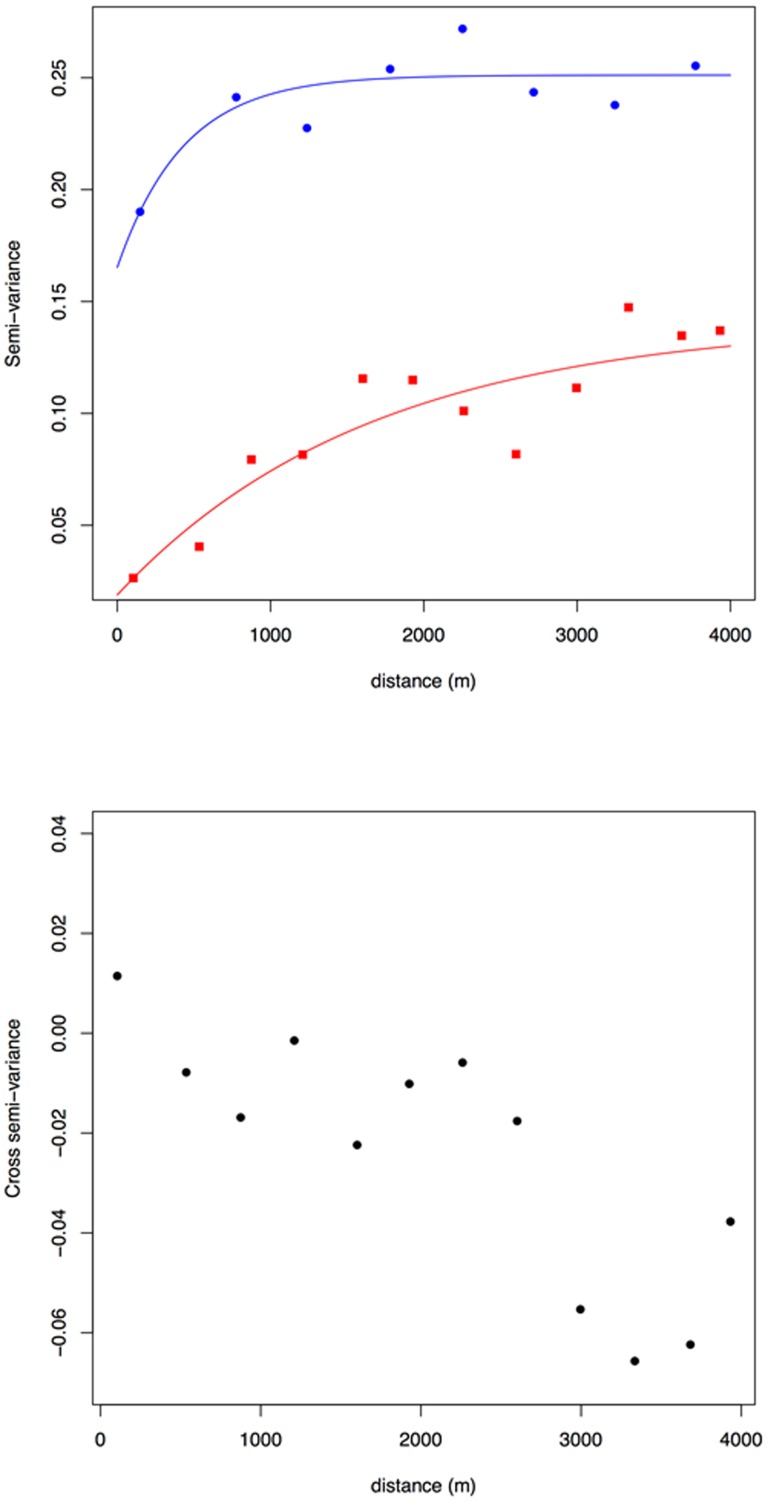
Geostatistical analysis of *M. natalensis* and *R. rattus* spatial distributions across the city of Niamey, Niger. (a) Indicator variograms for a threshold value Z_k_ = 0. Blue circles and red squares indicate empirical variograms for *M. natalensis* and *R. rattus*, respectively. Solid lines correspond to fitted exponential variogram models. (b) Cross indicator variogram for *M. natalensis* and *R. rattus* with a threshold value Z_k_ = 0.

The variograms showed that the indicator values for both *M. natalensis* and *R. rattus* exhibited a spatially structured distribution (Figure 3a). The semi-variance increased with distance up to a plateau (the sill in geostatistical jargon): this pattern indicated that neighboring samples tended to be more similar than expected under complete randomness. An experimental model was fitted to each empirical variogram (shown as solid lines in Figure 3a) and the corresponding parameters (not shown) were used in the kriging algorithm.

**Figure 4:**
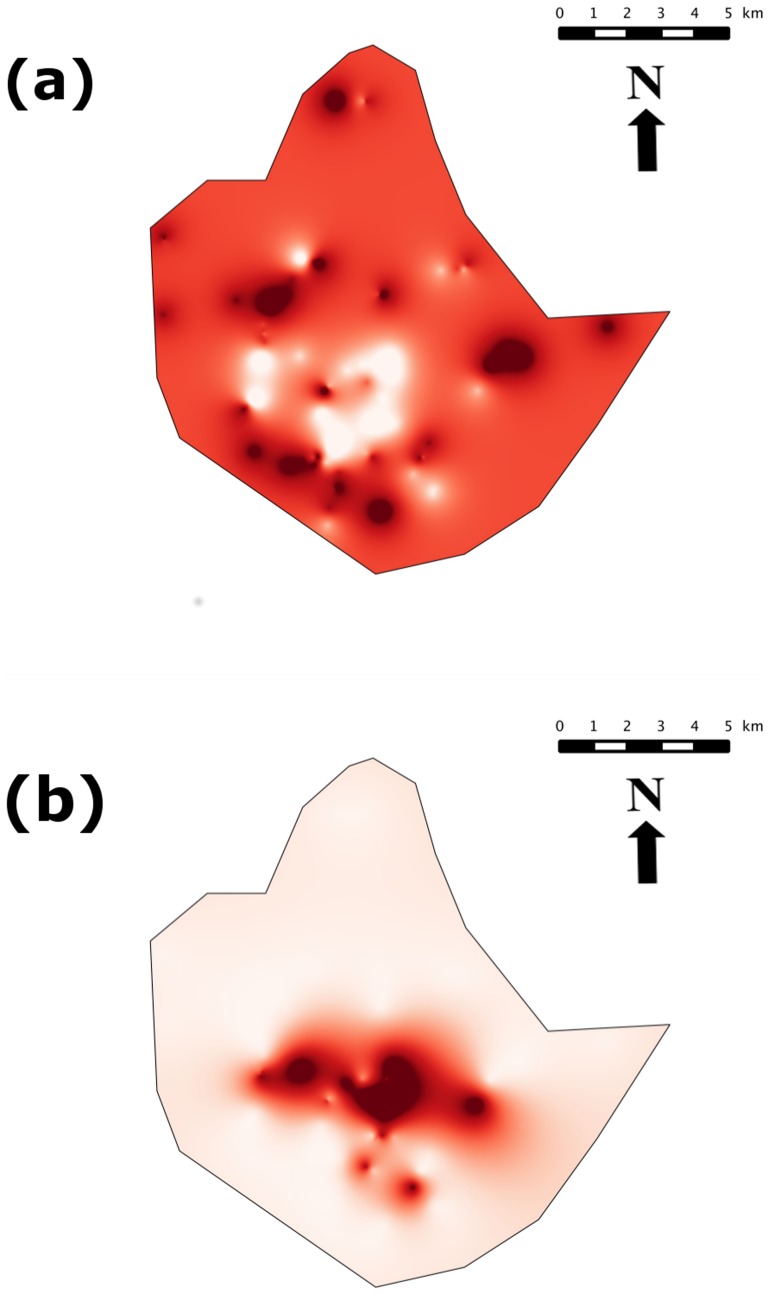
Indicator maps of *M. natalensis* (a) and *R. rattus* (b) spatial distribution across the city of Niamey (Niger). Mapped values were estimated by ordinary kriging using fitted indicator variograms for threshold value Z_k_ = 0. Values correspond to probabilities of observing abundances >0, i.e., probabilities of presence.

The range of the variograms (i.e., the distance at which the semi-variance reaches the plateau) indicated that the spatial dependence occurred at scales of ca. 400 and 1600 meters for *M. natalensis* and *R. rattus*, respectively. Ordinary kriging led to maps of probability given in Figure 4. The spatial distribution of *M. natalensis* showed the presence of large areas of high probability of detection as well as a central area where the probability was markedly lower (Figure 4a). The latter patches appeared to be short-scaled as it can be seen from Figure 4a. This is also indicated by the shape of the variogram which exhibited a short range. These results showed that *M. natalensis* was fairly homogeneously distributed across Niamey with scattered patches of high density in most areas of the city, except in the central part of town where the species was rare.

The spatial pattern of *R. rattus* was much strongly structured with more than 86% of spatial variance. It also corresponded to a larger range of ca. 1600 meters. The map (Figure 4b) shows that the probability of occurrence is high in the city center while it remains near-zero everywhere else.

This central area roughly corresponded to the range of the fitted variogram (Figure 3).

The comparison of *M. natalensis* and *R. rattus* maps suggested the presence of a spatial segregation between these species. This observation was confirmed by the shape of the cross-variogram (Figure 3b): the cross semi-variance decreased with increasing distance lag, thus conveying that the relationship between the two species faded away with increasing separating distance. Moreover, the cross semi-variance was negative: thus meaning that the species were negatively associated, *i.e.* they displayed spatial segregation.

## Discussion

Unlike surveys on rodent ecology from wild or rural areas, studies on urban rodents are quite scarce. When available, they usually deal with one single species (e.g., [22]) and/or with European (e.g., [16]), Asian (e.g., [18]) or American (e.g., [5], [20], [24]) cities. As far as we know, the only studies dedicated to urban rodent communities in Sub-Saharan Africa were conducted by Taylor and colleagues [19] in Durban, South Africa, and by Houéménou [52] in Cotonou, Benin.

Rodents are abundant and widespread in all built-up parts of Niamey since they were found in all 41 such localities and 75.3% (134 out of 178) of the human edifices (i.e., excluding gardens, fallow lands and rice-fields) investigated during the SP, as well as 100% (38 out of 38) of the trapping sites of the OP. As a comparison, the percentage of infested houses is 1–8% in the whole UK [16], but reaches 50% in the city of Manchester, UK [17], 32% of homes from poor areas of the US countryside [53], and 100% in farms of the Yucatan Province, Mexico [54]. Values between 46% and 58% were proposed for houses in rural areas in Senegal (Duplantier and Handschumcher, unpubl, quoted by [28]), although the latter ones may be underestimated since based only on visible damages that were *a posteriori* imputed to rodents (Duplantier, pers. comm.). We are aware of no such available data for urban areas elsewhere in Africa.

### 

#### Rodent species communities in Niamey: rural-like vs. urban assemblages

The rodent community observed in Niamey groups typical Sahelian taxa, including the two invasive species *M. musculus* and *R. rattus* which have already been recorded in many other West African towns and villages [28]. The total species richness reaches eight, thus representing 25% of that known for the whole country [31]. This value is similar to that found in Durban, South Africa (N = 7; [19]), but greater than that found in Makurdi, Nigeria (N = 4; [55]), although the latter survey has focused on habitations only, thus precluding the identification of sylvatic species.

From this perspective, the pattern observed in Niamey is quite interesting in that it shows a clear exclusion between two species assemblages: the first one (here below referred to as “rural-like species”) is found solely within inner-city cultivated areas (gardens and rice fields) and fallow lands (*A. niloticus*, *C. gambianus* and, most probably, *T. gracilis* – to which *X. erythropus* may be added), while the second one (designed below as “commensal urban species”) is made of truly commensal city-dwelling species (*M. natalensis*, *M. musculus* and *R. rattus*) that inhabits human-edified structures such as houses, ventures and shops, public buildings, factories, store rooms and markets. This tends to suggest that wild (hence native) species that once inhabited the local natural environment do not maintain in the newly urbanized environment where only true commensal species proliferate. This agrees with patterns observed in Buenos Aires where parklands and a surrounding natural reserve serves as a refuge for native taxa while only true commensal -and actually all invasive- species live in the industrial and residential part of the city [20]. However, in Niamey, one native species, namely *M. natalensis*, still co-exist with the two invasive *M. musculus* and *R. rattus* species within the core city. One should notice that *M. natalensis* is known as a truly commensal species all over West Africa where it seems perfectly adapted to human-edified structures [28]. In agreement with [20], this suggests that the highly modified habitat provided by cities acts as an “environmental buffer” where highly adaptable and competitive species (as expected for successful invasive ones; [56]) can proliferate without suffering from competition with locally adapted ones which have been co-evolving for a long time with the local but non-modified environment. From there, it is highly probable that only in those cases where a native species is already present and well adapted to human dwellings can a longstanding coexistence of commensal invasive and native species be expected within the urban habitat. Another African example may correspond to Cotonou, Benin, where house mice, black and Norway rats seem to coexist with the native *Mastomys* sp. in the city [52]).

As such, Niamey appears as an interesting case study where a native and truly commensal species is currently facing the recent and probably still ongoing invasions of two global commensal invaders [27].

#### Native vs. invasive species' interactions in the core city

Whatever the method considered, namely co-occurrence analysis, occupancy modelling and geostatistical approaches, species-specific co-distributions of truly commensal rodents clearly appear to be non-random in Niamey. Indeed, within the commensal urban species assemblage, we retrieved a strong trend towards an exclusive distribution of native (*M. natalensis*) and invasive (*M. musculus* and *R. rattus*) species with these three radically different methods. Actually, the overall pattern largely suggests that *M. natalensis* is distributed as a continuous layer throughout the core city, except in some plots where black rats and/or mice are present (Figures 2 and 4).

Interestingly, very high densities were locally found for both black rats (e.g., 93.6 ind/ha in CGA as inferred through instantaneous trapping success per surface area; see [27]) and mice (e.g., 253.8 ind/ha in GRM), and both species displayed obviously intensive reproduction: 25.5% (N = 41), 20.6% (N = 33) and 26.1% (N = 42) of the 161 black rats that could be typed for age and sexual activity were juveniles, gestating females and sexually active males, respectively, while 23.9% (N = 16) of the 67 typed mice were juveniles, 16.4% (N = 11) were gestating females and 29.9% (N = 20) were active males [27]. Altogether, such high densities and signs of active reproduction in both species strongly suggest (though do not demonstrate) that these two currently ongoing rodent invasions in Niamey will be successful since this probably reflects sustainable populations [57]. This would not really be unexpected when one considers how successful they are elsewhere in the World [7] including in neighbouring African countries (e.g., [52],[55],[58]–[59]; review in [28]).

We found both mice and rats essentially associated with urban areas that are characterized by intense commercial and exchange activities such as major markets, coach stations and stores, and that lies in the heart of town. Since rats and mice dispersal has already been associated with human transports in Africa [58], [60]–[61], it is reasonable to hypothesize that these species were imported to Niamey following people and goods exchanges. The native *M. natalensis* was found in many markets and industrial stores in Niger (Garba, Hima, unpubl.), including in Niamey (e.g., KAR, PEM, RTO in this study), thus confirming that the species is also able to inhabit this type of habitats. As a consequence, its absence in such places where black rats were found (e.g. ABA, KIR, J-CYA, GRM-M,) suggests that it was replaced by the invader. As a supporting argument, while most households shelter *M. natalensis* throughout Niamey, the massive colonization by mice of houses from the highly populated GRM district, together with the strict absence of *M. natalensis*, represent another clear illustration of such a native-to-invasive rodent species turn-over.

Altogether, our data show that the native species *M. natalensis* is widespread and abundant in all parts of the city, with the exception of some localized zones where it is replaced by the invasive species *M. musculus* and/or *R. rattus*. No clear socio-environmental factor could be detected to explain this marked invasive *vs*. native exclusion pattern (unpublished results; but see [27]). Keeping in mind the very recent history of the city of Niamey (see introduction), we believe that the exclusive distribution of native and invasive species is due to historical rather than ecological factors. In that case, the invasion would still be in progress and the current species distribution would only be partial with further potential expansion. This would explain why, at the time of our study, the invasive taxa would be mainly present within and around areas displaying important commercial exchange flows. From there, it could be predicted that mice and rats will progressively disperse in most if not all remaining parts of the town, as already suggested by the still scarce (though massively invaded) households that were identified as already invaded (mice in GRM; black rats in CYA and CGA). Such a scenario is also supported by what is now observed in several towns around the World where invasive rodents, essentially *R. rattus*, *R. norvegicus*, *R. exulans* and *M. musculus*, fully replaced native rodents, sometimes centuries ago (e.g., [8], [20], [58]–[59]).

This also raises the question about the nature of native/invasive species interactions underlying this turn-over. One hypothesis is that black rats or mice are more competitive than *M. natalensis* in the urban landscape of Niamey. In Mozambican villages, it has been observed that, when reaching high levels, *R. rattus* populations could regulate *M. natalensis'* ones [62], although the process at work remains unclear. This could be due to a better exploitation of resources and/or a reproductive advantage. In the same manner, it was shown that *M. natalensis* did not enter Tanzanian farms when black rats were present [63], thus pointing towards direct competition. In Niger, too, the two species are indeed found within the habitations, sometimes within the same rooms, thus suggesting that they indeed probably compete for food and space. Black rats were shown to be aggressive towards intruders with the physical elimination of experimentally introduced conspecific individuals within an insular population [64]. However, *M. natalensis* can also be very aggressive towards other congeneric species (see *Mastomys* sp.3, in [65]). Unfortunately, we are aware of no study that focused on *M. natalensis*/*R. rattus* interactions in the wild, *a fortiori* in an urban environment where densities may be high. If one considers adults' external measurements, black rats from Africa appear to be much larger than *Mastomys* (males: 99 g vs. 51.9 g, respectively; females: 94.8 g vs. 49.9 g; [28]), and one may expect the former to physically eliminate the latter. However, no proof of this exists, and size can clearly not be advocated for the replacement of *M. natalensis* by mice which are much smaller (13.3 g in males, 13.2 g in females; [28]).

Another explanation relies on reproduction, with black rats and mice showing higher reproductive capacities than the native species. Yet, this seems poorly credible since *M. natalensis* is actually the most prolific, with 21–22 days long gestation, an average litter size of 6 [28], an average of 8.4 embryos per gestating female (based on 129 individuals from Niamey; Garba and Dobigny, unpubl.) and permanent reproduction all year long in the city [66]. As a comparison, black rats display 20–22 days long gestation, 5.4 cubs per litter (data from West Africa; [28]) and 5.9 embryos per female (based on 24 individuals from Niamey; Garba and Dobigny, unpubl.), while domestic mice are characterized by 19–20 days long gestation (data from West Africa; [28]) and 4.1 embryos per female (based on 11 individuals from Niamey; Garba and Dobigny, unpubl.).

Finally, a third and non-exclusive hypothesis involving parasites would deserve to be scrutinized. Indeed, black rats and domestic mice may introduce new parasites that could jump to *M. natalensis*. Some of them may be much more virulent for the native (hence naïve) rodents than for the original reservoirs which have undergone long co-evolutionary interactions with these pathogens. This would clearly provide a strong advantage to the invasive organism. Such a process, known as “spill-over” [67], has already been shown to involve black rats, as in the case of *R. rattus* that were introduced to Christmas Island while carrying *Trypanosoma lewisi*, a pathogen that ultimately decimated all indigenous rodents [68]–[69]. Interestingly, the same *T. lewisi* has been detected in black rats from South-Western Niger [70] as well as in Niamey (our own unpublished data) but it remains to see whether that will have any effects on rodent species composition.

#### Societal implications and perspectives

Our study shows that rodents are widespread and abundant in Niamey, especially in the core city where infestation rates are high. This probably translates into important nuisances, as supported by local perception by inhabitants: 96% of the 170 interviewed persons mentioned rodent-associated troubles at home (damages on food and stocks, houses and furniture, etc; [71]). Importantly, in no instance were potential health problems cited by people [71]. Yet, several human diseases involving rodents exist in Niger (e.g., leishmaniases: [72]; toxoplasmosis: [73]). Moreover, our study highlights the important changes in urban rodent assemblages that are most probably to come in Niamey, with at least two recent bioinvasions that involve black rats and domestic mice. Both organisms are well known for their major role in the maintaining and circulation of many human pathogens (reviews in [14]–[15]). The import of the allochtonous rat-borne *Trypanosoma lewisi* in South Western Niger [70] is a concrete example of possible consequences that these processes may have on public health since this parasite has already been shown to induce human death in Senegal [74]. We believe that this is particularly important to keep in mind since we predict that black rats and mice have the potential to spread further in Niger, maybe until complete replacement of native rodent communities as observed in other places in the World (see above). If this was to happen, induced modifications of rodent-borne pathogens communities would probably be drastic, including introduction and dissemination of new pathogens, recombination between local and newly introduced strains, etc.

Finally, we also think that the absence of *R. norvegicus* deserves to be highlighted since this other invasive species has been mentioned from neighbouring countries such as Senegal, Mali, Benin [28], [52], [75] as well as several areas from Nigeria (e.g., [55], [76]–[77]), but it may not have reached Niger yet [27, 31, this study]. If this was to occur, interactions between the two rat species would be hardly predictable since, when the two species coexist, the Norway rat tends to replace the black one in many cities (e.g., [13], [19]) though not all (e.g., [20]). It has also been suggested that *R. rattus* may be better adapted to warm climates than is *R. norvegicus* which rather settles in temperate to cold ones [20], [23], [78]. Yet, Norway rats seem to be highly successful in Bamako city and in the “Office du Niger” irrigation scheme in Mali (Dalecky et al., in prep.) which provides very different environmental but similar climatic conditions compared to Niamey. This suggests that climate alone may not be sufficient to explain rats' distribution and potential range of invasion. Clearly, historical and urban landscape characteristics of each city and its associated exchange network are other important elements to take into consideration when predicting invasion ranges dynamics and invasion risk.

## Supporting Information

Table S1 **Trapping-based inference of detection/non-detection as well as probability of detection in each of the 166 SP trapping sites investigated by occupancy modelling.** Trapping sites are labelled according to a “C-XXX-N” code (where “XXX” refers to a locality and “N” is an unique number within this given locality), except “ABA” and “KIR” which correspond to the two industrial sites (see text for details).
(DOCX)

Table S2 **Model selection results for** ***Rattus rattus*** **,** ***Mus musculus*** **, and** ***Mastomys natalensis*** **, where ψ is the probabilities of presence and** ***p*** **is the probability of detection of the species in a trap.** Models are compared with ΔAIC, Deviance and Akaike weight (*w*). *k* indicates the number of parameters of the model. Covariates used in the model are *i* for a constant parameter, and *trap* for a detection parameter varying depending on the type of traps.
(DOCX)
