# Supplementary material for: Spatial Segregation between Invasive and Native Commensal Rodents in an Urban Environment: A Case Study in Niamey, Niger
Source: PLoS One. 2014 Nov 7;9(11):e110666. doi: 10.1371/journal.pone.0110666 (PMC4224371; doi:10.1371/journal.pone.0110666)
Supplement: Table S1 — Trapping-based inference of detection/non-detection as well as probability of detection in each of the 166 SP trapping sites investigated by occupancy modelling. Trapping sites are labelled according to a “C-XXX-N” code (where “XXX” refers to a locality and “N” is an unique number within this given locality), except “ABA” and “KIR” which correspond to the two industrial sites (see text for details). (DOCX) [file pone.0110666.s001.docx]

**Table S1**

|  | Trapping-based assessment of presence/absence | | | Occupancy model-based probability of presence | | |
| --- | --- | --- | --- | --- | --- | --- |
| Trapping site | *M. natalensis* | *R. rattus* | *M. musculus* | *M. natalensis* | *R. rattus* | *M. musculus* |
|  |  |  |  |  |  |  |
| ABA | 0 | 1 | 0 | 0 | 1 | 0 |
| C-BAF2-1 | 1 | 0 | 0 | 1 | 0 | 0 |
| C-BAF2-2 | 0 | 0 | 0 | 0,6462 | 0,0316 | 0,0107 |
| C-BAF2-3 | 1 | 0 | 0 | 1 | 0,0074 | 0,0002 |
| C-BAF2-4 | 0 | 0 | 0 | 0,2001 | 0,0074 | 0,0002 |
| C-BAF2-5 | 0 | 0 | 0 | 0,6462 | 0,0316 | 0,0107 |
| C-BAF2-6 | 0 | 0 | 0 | 0,4849 | 0,0195 | 0,0026 |
| C-BAF2-7 | 0 | 0 | 0 | 0,6462 | 0,0316 | 0,0107 |
| C-BAF2-8 | 1 | 0 | 0 | 1 | 0,0047 | 0,0001 |
| C-BAF2-9 | 1 | 0 | 0 | 1 | 0,008 | 0,0003 |
| C-BOU-1 | 1 | 0 | 0 | 1 | 0,001 | 0 |
| C-BOU-2 | 1 | 0 | 0 | 1 | 0,0212 | 0,0053 |
| C-BOU-3 | 1 | 0 | 0 | 1 | 0,012 | 0,0006 |
| C-BOU-4 | 0 | 0 | 0 | 0,3267 | 0,012 | 0,0006 |
| C-BOU-5 | 0 | 0 | 0 | 0,4849 | 0,0195 | 0,0026 |
| C-BOU-6 | 1 | 0 | 0 | 1 | 0 | 0 |
| C-BOU-7 | 1 | 0 | 0 | 1 | 0,0009 | 0 |
| C-BOU-8 | 1 | 0 | 0 | 1 | 0,0001 | 0 |
| C-CGA-1 | 0 | 0 | 0 | 0,2239 | 0,0009 | 0,0006 |
| C-CGA-2 | 0 | 1 | 0 | 0,1849 | 1 | 0,0002 |
| C-CGA-3 | 0 | 0 | 0 | 0,2001 | 0,0074 | 0,0002 |
| C-CGA-4 | 1 | 1 | 0 | 1 | 1 | 0 |
| C-CGA-5 | 0 | 0 | 0 | 0,0623 | 0,0028 | 0 |
| C-CGA-6 | 0 | 0 | 0 | 0,4849 | 0,0195 | 0,0026 |
| C-CGA-7 | 0 | 0 | 0 | 0,3267 | 0,012 | 0,0006 |
| C-CGA-8 | 0 | 0 | 0 | 0,3267 | 0,012 | 0,0006 |
| C-CGA-9 | 0 | 0 | 0 | 0,1142 | 0,0045 | 0 |
| C-CYA-1 | 1 | 0 | 0 | 1 | 0,0102 | 0,0002 |
| C-CYA-10 | 0 | 1 | 0 | 0,2988 | 1 | 0,0006 |
| C-CYA-11 | 0 | 0 | 0 | 0,1142 | 0,0045 | 0 |
| C-CYA-12 | 0 | 0 | 0 | 0,2913 | 0,0107 | 0,0005 |
| C-CYA-13 | 0 | 0 | 0 | 0,4849 | 0,0195 | 0,0026 |
| C-CYA-14 | 1 | 0 | 0 | 1 | 0,0195 | 0,0026 |
| C-CYA-2 | 1 | 0 | 0 | 1 | 0,0003 | 0 |
| C-CYA-3 | 1 | 0 | 0 | 1 | 0,0195 | 0,0026 |
| C-CYA-4 | 1 | 0 | 0 | 1 | 0,012 | 0,0006 |
| C-CYA-5 | 1 | 0 | 0 | 1 | 0,0111 | 0,0003 |
| C-CYA-6 | 1 | 0 | 0 | 1 | 0,0013 | 0 |
| C-CYA-7 | 1 | 0 | 0 | 1 | 0,0045 | 0 |
| C-CYA-8 | 1 | 0 | 0 | 1 | 0,0045 | 0 |
| C-CYA-9 | 1 | 0 | 0 | 1 | 0,0017 | 0 |
| C-DAR-1 | 1 | 0 | 0 | 1 | 0,0001 | 0 |
| C-DAR-2 | 1 | 0 | 0 | 1 | 0 | 0 |
| C-DAR-3 | 0 | 0 | 0 | 0,6312 | 0,0229 | 0,0107 |
| C-DAR-4 | 1 | 0 | 0 | 1 | 0,0002 | 0 |
| C-DAR-5 | 1 | 0 | 0 | 1 | 0,0045 | 0 |
| C-DAR-6 | 1 | 0 | 0 | 1 | 0,0316 | 0,0107 |
| C-DAR-7 | 1 | 0 | 0 | 1 | 0,0074 | 0,0002 |
| C-DAR-8 | 1 | 0 | 0 | 1 | 0,0002 | 0 |
| C-GAM-1 | 0 | 0 | 0 | 0,1617 | 0,002 | 0,0002 |
| C-GAM-10 | 0 | 0 | 0 | 0,0173 | 0,001 | 0 |
| C-GAM-11 | 1 | 0 | 0 | 1 | 0,0195 | 0,0026 |
| C-GAM-12 | 1 | 0 | 0 | 1 | 0,0195 | 0,0026 |
| C-GAM-13 | 1 | 0 | 0 | 1 | 0,0316 | 0,0107 |
| C-GAM-2 | 0 | 0 | 0 | 0,3412 | 0,0166 | 0,0006 |
| C-GAM-3 | 1 | 0 | 0 | 1 | 0,0045 | 0 |
| C-GAM-4 | 1 | 0 | 0 | 1 | 0,0003 | 0 |
| C-GAM-5 | 1 | 0 | 0 | 1 | 0,0074 | 0,0002 |
| C-GAM-6 | 1 | 0 | 0 | 1 | 0,0195 | 0,0026 |
| C-GAM-7 | 0 | 0 | 0 | 0,3267 | 0,012 | 0,0006 |
| C-GAM-8 | 1 | 0 | 0 | 1 | 0,0074 | 0,0002 |
| C-GAM-9 | 1 | 0 | 0 | 1 | 0,0014 | 0 |
| C-GNA-1 | 1 | 0 | 0 | 1 | 0,0195 | 0,0026 |
| C-GNA-10 | 1 | 0 | 0 | 1 | 0,0006 | 0 |
| C-GNA-11 | 1 | 0 | 0 | 1 | 0,0195 | 0,0026 |
| C-GNA-2 | 1 | 0 | 0 | 1 | 0,003 | 0 |
| C-GNA-3 | 1 | 0 | 0 | 1 | 0,0141 | 0,0026 |
| C-GNA-4 | 1 | 0 | 0 | 1 | 0,0045 | 0 |
| C-GNA-5 | 1 | 0 | 0 | 1 | 0,0195 | 0,0026 |
| C-GNA-6 | 1 | 0 | 0 | 1 | 0,0004 | 0 |
| C-GNA-7 | 0 | 0 | 0 | 0,2107 | 0,0102 | 0,0002 |
| C-GNA-8 | 1 | 0 | 0 | 1 | 0,0074 | 0,0002 |
| C-GNA-9 | 1 | 0 | 0 | 1 | 0,0074 | 0,0002 |
| C-GRM-1 | 0 | 1 | 1 | 0,0047 | 1 | 1 |
| C-GRM-2 | 0 | 0 | 1 | 0,2001 | 0,0074 | 1 |
| C-GRM-3 | 0 | 1 | 1 | 0,4687 | 1 | 1 |
| C-GRM-4 | 0 | 0 | 1 | 0,4849 | 0,0195 | 1 |
| C-GRM-5 | 0 | 0 | 1 | 0,003 | 0,0003 | 1 |
| C-GRM-6 | 0 | 0 | 1 | 0,5222 | 0,0203 | 1 |
| C-GRM-7 | 0 | 0 | 1 | 0,0353 | 0,0024 | 1 |
| C-KAR-10 | 1 | 0 | 0 | 1 | 0,0019 | 0 |
| C-KAR-11 | 1 | 0 | 0 | 1 | 0,004 | 0 |
| C-KAR-12 | 1 | 0 | 0 | 1 | 0,004 | 0 |
| C-KAR-13 | 1 | 0 | 0 | 0,0635 | 0,0015 | 0 |
| C-KAR-14 | 1 | 0 | 0 | 1 | 0,0166 | 0,0006 |
| C-KAR-15 | 1 | 0 | 0 | 1 | 0,0125 | 0,0009 |
| C-KAR-16 | 1 | 0 | 0 | 1 | 0,0047 | 0,0001 |
| C-KAR-17 | 1 | 0 | 0 | 1 | 0,0074 | 0,0002 |
| C-KAR-18 | 0 | 0 | 0 | 0,4849 | 0,0195 | 0,0026 |
| C-KAR-19 | 1 | 0 | 0 | 1 | 0,0031 | 0 |
| C-KAR-2 | 0 | 0 | 0 | 0,5673 | 0,0249 | 0,0053 |
| C-KAR-20 | 1 | 0 | 0 | 1 | 0,013 | 0,0013 |
| C-KAR-21 | 1 | 0 | 0 | 1 | 0,0074 | 0,0002 |
| C-KAR-22 | 0 | 0 | 0 | 0,4849 | 0,0195 | 0,0026 |
| C-KAR-23 | 1 | 0 | 0 | 0,4849 | 0,0195 | 0,0026 |
| C-KAR-24 | 1 | 0 | 0 | 1 | 0,0077 | 0,0002 |
| C-KAR-3 | 1 | 0 | 0 | 1 | 0,0249 | 0,0053 |
| C-KAR-5 | 1 | 0 | 0 | 1 | 0,0249 | 0,0053 |
| C-KAR-6 | 1 | 0 | 0 | 0,5553 | 0,0195 | 0,0053 |
| C-KAR-7 | 1 | 0 | 0 | 1 | 0,0173 | 0,0019 |
| C-KAR-8 | 1 | 0 | 0 | 1 | 0,012 | 0,0006 |
| C-KOT-1 | 0 | 0 | 0 | 0,5855 | 0,0026 | 0,0107 |
| C-KOT-2 | 0 | 0 | 0 | 0,3267 | 0,012 | 0,0006 |
| C-KOT-3 | 0 | 0 | 0 | 0,6462 | 0,0316 | 0,0107 |
| C-KOT-4 | 1 | 0 | 0 | 1 | 0,0003 | 0 |
| C-KOT-5 | 1 | 0 | 0 | 1 | 0,0007 | 0 |
| C-KOT-6 | 1 | 0 | 0 | 1 | 0,012 | 0,0006 |
| C-KOT-7 | 1 | 0 | 0 | 1 | 0,0195 | 0,0026 |
| C-KOU-1 | 1 | 0 | 0 | 1 | 0,0028 | 0 |
| C-KOU-10 | 1 | 0 | 0 | 1 | 0,0045 | 0 |
| C-KOU-11 | 1 | 0 | 0 | 1 | 0,0316 | 0,0107 |
| C-KOU-12 | 1 | 0 | 0 | 1 | 0,0195 | 0,0026 |
| C-KOU-2 | 0 | 0 | 0 | 0,0173 | 0,001 | 0 |
| C-KOU-3 | 0 | 0 | 0 | 0,2001 | 0,0074 | 0,0002 |
| C-KOU-4 | 1 | 0 | 0 | 1 | 0,012 | 0,0006 |
| C-KOU-5 | 1 | 0 | 0 | 1 | 0,0074 | 0,0002 |
| C-KOU-6 | 0 | 0 | 0 | 0,3267 | 0,012 | 0,0006 |
| C-KOU-7 | 1 | 0 | 0 | 1 | 0,0077 | 0,0002 |
| C-KOU-8 | 1 | 0 | 0 | 1 | 0,0045 | 0 |
| C-KOU-9 | 1 | 0 | 0 | 1 | 0,012 | 0,0006 |
| C-LMO-1 | 1 | 0 | 0 | 1 | 0,0195 | 0,0026 |
| C-LMO-2 | 1 | 0 | 0 | 1 | 0 | 0 |
| C-LMO-3 | 1 | 0 | 0 | 1 | 0,0003 | 0 |
| C-LMO-4 | 1 | 0 | 0 | 1 | 0,0074 | 0,0002 |
| C-LMO-5 | 1 | 0 | 0 | 1 | 0,0017 | 0 |
| C-LMO-6 | 0 | 0 | 0 | 0,6462 | 0,0316 | 0,0107 |
| C-LMO-7 | 1 | 0 | 0 | 1 | 0,0269 | 0,0026 |
| C-PKE-1 | 1 | 0 | 0 | 1 | 0,0004 | 0 |
| C-PKE-2 | 0 | 0 | 0 | 0,4525 | 0,0102 | 0,0026 |
| C-PKE-3 | 1 | 0 | 0 | 1 | 0,0011 | 0 |
| C-PKE-4 | 1 | 0 | 0 | 1 | 0,0074 | 0,0002 |
| C-PKE-5 | 0 | 0 | 0 | 0,0331 | 0,0017 | 0 |
| C-PKE-6 | 1 | 0 | 0 | 1 | 0,0001 | 0 |
| C-PKE-7 | 1 | 0 | 0 | 1 | 0,037 | 0,0026 |
| C-PKE-8 | 1 | 0 | 0 | 1 | 0,0195 | 0,0026 |
| C-PKE-9 | 1 | 0 | 0 | 1 | 0,0028 | 0 |
| C-ROF-1 | 1 | 0 | 0 | 1 | 0 | 0 |
| C-ROF-2 | 1 | 0 | 0 | 1 | 0 | 0 |
| C-ROF-3 | 1 | 0 | 0 | 1 | 0,0195 | 0,0026 |
| C-ROF-4 | 1 | 0 | 0 | 1 | 0,0094 | 0,0001 |
| C-TCH-1 | 1 | 0 | 0 | 1 | 0,002 | 0,0002 |
| C-TCH-2 | 1 | 0 | 0 | 1 | 0,0023 | 0,0107 |
| C-TCH-3 | 1 | 0 | 0 | 1 | 0,0074 | 0,0026 |
| C-TCH-4 | 1 | 0 | 0 | 1 | 0,0017 | 0 |
| C-TCH-5 | 1 | 0 | 0 | 1 | 0,0017 | 0 |
| C-WAD-1 | 1 | 0 | 0 | 1 | 0 | 0 |
| C-WAD-2 | 0 | 0 | 0 | 0,3412 | 0,0166 | 0,0006 |
| C-WAD-3 | 1 | 0 | 0 | 1 | 0,001 | 0 |
| C-WAD-4 | 1 | 0 | 0 | 1 | 0,0011 | 0 |
| C-WAD-5 | 1 | 0 | 0 | 1 | 0,0005 | 0 |
| C-WAD-6 | 1 | 0 | 0 | 1 | 0,0001 | 0 |
| C-WAD-7 | 1 | 0 | 0 | 1 | 0,0022 | 0 |
| C-YAB-1 | 0 | 0 | 0 | 0,0013 | 0,0002 | 0 |
| C-YAB-10 | 1 | 0 | 0 | 1 | 0,001 | 0,0002 |
| C-YAB-11 | 0 | 0 | 0 | 0,0331 | 0,0017 | 0 |
| C-YAB-12 | 1 | 0 | 0 | 1 | 0,0074 | 0,0002 |
| C-YAB-2 | 0 | 0 | 0 | 0,5011 | 0,0269 | 0,0026 |
| C-YAB-3 | 1 | 0 | 0 | 1 | 0,0045 | 0,0006 |
| C-YAB-4 | 1 | 0 | 0 | 1 | 0,0002 | 0 |
| C-YAB-5 | 1 | 0 | 0 | 1 | 0,0269 | 0,0026 |
| C-YAB-6 | 1 | 0 | 0 | 1 | 0,0195 | 0,0026 |
| C-YAB-7 | 0 | 0 | 0 | 0,5222 | 0,0203 | 0,0038 |
| C-YAB-8 | 0 | 0 | 0 | 0,4849 | 0,0195 | 0,0026 |
| C-YAB-9 | 1 | 0 | 0 | 1 | 0,013 | 0,0013 |
| C-YAH-3 | 1 | 0 | 0 | 1 | 0,0014 | 0 |
| C-YAH-5 | 1 | 0 | 0 | 1 | 0,0045 | 0 |
| C-YAH-6 | 1 | 0 | 0 | 1 | 0,0001 | 0 |
| C-YAH-7 | 1 | 0 | 0 | 1 | 0,012 | 0,0006 |
| C-YAH-8 | 1 | 0 | 0 | 1 | 0,0074 | 0,0002 |
| C-YAH-9 | 0 | 0 | 0 | 0,6462 | 0,0316 | 0,0107 |
| KIR | 0 | 1 | 0 | 0 | 1 | 0 |
