# Supplementary material for: Spatial Segregation between Invasive and Native Commensal Rodents in an Urban Environment: A Case Study in Niamey, Niger
Source: PLoS One. 2014 Nov 7;9(11):e110666. doi: 10.1371/journal.pone.0110666 (PMC4224371; doi:10.1371/journal.pone.0110666)
Supplement: Table S2 — Model selection results for Rattus rattus , Mus musculus , and Mastomys natalensis , where ψ is the probabilities of presence and p is the probability of detection of the species in a trap. Models are compared with ΔAIC, Deviance and Akaike weight (w). k indicates the number of parameters of the model. Covariates used in the model are i for a constant parameter, and trap for a detection parameter varying depending on the type of traps. (DOCX) [file pone.0110666.s002.docx]

Table S2

| ψ | *p* | ΔAIC | *w* | *k* | Deviance |
| --- | --- | --- | --- | --- | --- |
|  |  |  |  |  |  |
| *Rattus rattus* | | | | | |
| *i* | *trap* | 0 | 0.952 | 3 | 573 |
| *i* | *i* | 6 | 0.048 | 2 | 581 |
| *Mus musculus* | | | | | |
| *i* | *trap* | 0 | >0.999 | 3 | 310 |
| *i* | *i* | 68 | <0.001 | 2 | 240 |
| *Mastomys natalensis* | | | | | |
| *i* | *trap* | 0 | >0.999 | 3 | 2898 |
| *i* | *i* | 513 | <0.001 | 2 | 3413 |
